# Editorial: Phage-based interventions in livestock: from genomics to translational applications

**DOI:** 10.3389/fmicb.2026.1944669

**Published:** 2026-08-17

**Authors:** Sonia Zapata-Mena, Christian Vinueza-Burgos, William Calero-Cáceres

**Affiliations:** 1 Instituto de Microbiología, Colegio de Ciencias Biológicas y Ambientales, Universidad San Francisco de Quito USFQ, Quito, Ecuador; 2 Unidad de Investigación de Enfermedades Transmitidas por Alimentos y Resistencia a los Antimicrobianos (UNIETAR), Facultad de Medicina Veterinaria y Zootecnia, Universidad Central del Ecuador, Quito, Ecuador; 3 Biotechnology Program, Department of Food and Biotechnology Science and Engineering, Universidad Técnica de Ambato, Ambato, Ecuador; 4 UTA-RAM-One Health, Group for Universal Advance in Bioscience, Department of Food and Biotechnology Science and Engineering, Universidad Técnica de Ambato, Ambato, Ecuador

**Keywords:** genomics, livestock, omics, phage therapy, phage (bacteriophage)

## Introduction

Animal production systems are simultaneously essential to global food security; however, the extensive use of antimicrobials in these systems has significantly contributed to the emergence and spread of antimicrobial resistance (AMR) (Clifford Astbury et al., 2025; Stehling et al., 2023). Food-producing animals account for most of the global antimicrobial consumption, sustaining selective pressure across food-producing animal sectors (Schar et al., 2020; Van Boeckel et al., 2015). This pressure has selected multidrug-resistant *Escherichia coli, Salmonella enterica, Campylobacter* spp., *Streptococcus* spp., and other pathogens in animal, human, and environmental reservoirs (Izydorcyzk and Church, 2026; Tiseo et al., 2020). As the antibiotic pipeline fails to keep pace, bacteriophages have re-emerged as one of the most credible alternatives for veterinary use (Calero-Cáceres and Balcázar, 2025; Topa-Pila et al., 2026).

Yet the path from an isolated phage to a validated veterinary product is long. It requires well-characterized phages and lysins, rational cocktail design, an understanding of prophage ecology and horizontal gene transfer, evolution-informed strategies to manage resistance, curated phage biobanks that enable precision selection, and regulatory frameworks (Bhale et al., 2026; Calero-Cáceres et al., 2026; Kaneko et al., 2026; Melaku et al., 2025; Mora-Domínguez and Calero-Cáceres, 2025).

This Research Topic assembles twelve contributions that span this full arc: novel phage and endolysin discovery for animal-associated pathogens, prophage genomic surveillance, evolution-informed phage engineering, alternative *in vivo* models, species-focused translational syntheses, and integrative One Health roadmaps.

## Discovery and characterization of novel phages and endolysins for animal-associated pathogens

Four original studies expand the phage and lysin repertoire against economically and zoonotically important pathogens of animal production. Wen et al. isolated phage WEN7 from hospital wastewater using a multidrug-resistant *Pseudomonas aeruginosa* PAO1-SZ1 host; the phage lysed 19 of 29 clinical *P. aeruginosa* isolates (65.5%), showed a burst size of ~165 PFU/cell and a 66,379 bp genome free of resistance and virulence determinants, remained stable across temperature, pH, UV, and ethanol stress, and displayed bactericidal activity in both milk and hospital wastewater—matrices that bridge clinical, food-safety, and environmental deployment. Zhao et al. characterized phage vB_RanS_GDF21 targeting *Riemerella anatipestifer*, the etiological agent of duck infectious serositis; the phage carries a 46,925 bp genome free of resistance and virulence genes, and its recombinant endolysin LysGDF21 both inhibited biofilm formation and disrupted mature biofilms, showing broad antibacterial activity across the *R. anatipestifer* panel. Shang et al. identified Ply691, a prophage-encoded lysin from *Streptococcus suis* SC267 with an unusual four-domain architecture (N-terminal Amidase-5, two central CW-7 binding domains, C-terminal Glucosaminidase), temperature adaptability from 4 to 37 °C and alkaline tolerance across pH 7–10, lytic activity against 11 *S. suis* serotypes, and, in a murine bacteremia model, 100% survival at 2 mg per mouse administered intraperitoneally 1 h post-infection, with substantial reduction of bacterial burden across major organs and no detectable resistance emergence. Lu et al. isolated and genomically characterized *Myoviridae* phage LQ5 (171,908 bp, carrying endolysin and holin lysis-mediator genes) against avian pathogenic *E. coli* (APEC) serotype O78; in a chick challenge model, phage administration reduced hepatic and splenic bacterial loads more effectively than amoxicillin.

## Prophage ecology and evolution-informed phage design

Two contributions turn to the bacterial genome itself as both a source of information and a substrate for engineering. Quelal-Madrid et al. performed the first systematic characterization of prophage diversity in 142 *S. enterica* genomes from poultry and clinical sources, using Phigaro, PHASTEST, and a terminase-based custom BLAST database. They found *Peduovirus* pro483 prevalent in *S*. Infantis, distinct cytosine-specific methyltransferase-bearing prophages in related *S*. Enteritidis, and Enterobacteria phage ST104 in *S*. Typhimurium encoding SieA and SieB superinfection-exclusion proteins that may confer competitive advantage within microbial communities. The study positions prophages as active drivers of serovar adaptation, virulence potential, and phage-resistance in a Latin American poultry system, and highlights that any deployed lytic-phage cocktail must be designed with awareness of the resident prophage landscape.

Luo et al. take an explicitly evolution-informed approach by developing a sequential positive–negative selection strategy—omitting conventional liquid enrichment to preserve viral diversity—to isolate two TolC-dependent phages, PTolC-28 and PTolC-69, directly from poultry-farm environments. Both exploit the outer-membrane exit duct of the AcrAB–TolC multidrug efflux pump as their receptor, enabling two distinct therapeutic modes. PTolC-28 drives “phage steering” in a strain-specific manner: in isolate GDW21C03, phage escape was mediated by a >60% reduction in *tolC* mRNA (rather than coding-sequence mutation), producing collateral re-sensitization to fluoroquinolones (~5.3-fold MIC drop), tetracyclines, and aminoglycosides. PTolC-69, in contrast, exhibited robust phage–antibiotic synergy with doxycycline and florfenicol, reducing effective antibiotic dose 8-fold *in vitro* and, in a chick infection model, reducing lung and spleen bacterial loads by nearly two orders of magnitude relative to either agent alone.

## Bridging the laboratory–farm gap: intermediate models and combination therapies

Translating phage efficacy from bench to production animal is limited by ethical, economic, and methodological constraints of vertebrate studies. Ruiz-Santamaría et al. address this bottleneck for *Campylobacter*, the leading cause of foodborne zoonosis in the EU, by validating *Galleria mellonella* as a bridging host for phage evaluation. Their four-phage cocktail lysed all 13 tested *Campylobacter* strains *in vitro*. *In vivo* virulence screening revealed marked strain dependence—only five of 13 strains reduced larval survival below 50%—and efficacy was optimized on the most virulent isolate, *C. jejuni* CJE065. At a multiplicity of infection (MOI) of 10, the cocktail raised larval survival from 25.5 to 57.5% (*p* < 0.001), and combination with erythromycin or ciprofloxacin further increased survival to 88.8% and 83.8%, respectively (*p* < 0.001). Beyond specific efficacy data, the study establishes an invertebrate screening platform that reduces reliance on vertebrate trials while enabling systematic optimization of dosing and phage–antibiotic combinations before farm-scale validation.

## Species-focused translational syntheses

Three reviews synthesize the state of phage-based intervention across biologically and epidemiologically distinct animal systems. Xu et al. comprehensively review phage-based interventions in livestock, poultry, and aquaculture, framing modern phage strategies along three mechanistic axes—direct bacterial killing, immune modulation, and antigen delivery—and distinguishing lytic phage therapy from phage-display-derived interventions and CRISPR-Cas-enabled theranostic platforms. Beyond cataloging applications, they critically examine translational bottlenecks (host-range constraints, pharmacokinetics, microbiota effects, and regulatory fragmentation) and openly note that most *in vivo* evidence remains preclinical.

Jespersen et al., in a mini-review, focus on *Salmonella* Dublin—a host-adapted, zoonotic, and characteristically intracellular bovine pathogen with strong regional clonality. They argue that this clonality is itself a design advantage for geographically tailored cocktails, while cataloging the persistent gaps in receptor knowledge, phage co-evolution data, and rumen-compatible administration routes. They also address a distinctive constraint for this serotype: because *S*. Dublin survives and replicates inside macrophages, effective phage therapy will depend on phages that internalize efficiently, a property that varies with phage size and morphotype.

Walter et al. present a PRISMA-guided systematic review of phages against *Paenibacillus larvae*, the causative agent of American foulbrood: from 90 initial records, nine studies met inclusion (three at the adult-bee or colony level, four in larvae, and two *in vitro*), collectively characterizing 27 phages *in vitro*, 18 in larvae, and nine at hive scale. In a controlled hive trial, a three-phage cocktail provided 100% protection vs. 80% infection in controls (*p* < 0.05), with full recovery within 2 weeks and performance comparable to or better than prophylactic antibiotics. Yet a biodistribution study documented only ~32 PFU per larva reaching brood 24 h after adult feeding, identifying hive-mediated inactivation—not intrinsic phage efficacy—as the dominant translational barrier. Notably, one phage was shown to bind both vegetative cells and spores while retaining ~93% of its *in vitro* infectivity, an unusually favorable property for a spore-forming pathogen and a mechanistic lead for future formulation.

## Frameworks for One Health deployment

Two contributions provide the integrative scaffolding needed to move from individual proofs-of-concept toward coordinated deployment. Atterbury et al., writing on behalf of the STAR-IDAZ international research consortium's Alternatives to Antimicrobials working group, present a roadmap for the veterinary and One Health application of phage technology, laying out the sequential requirements: phage selection, phage–host interaction characterization, disease- and species-specific application, One Health impact assessment, regulatory adaptation, and rational combination with other therapies. Cuteri et al. examine how artificial intelligence could accelerate each node of that roadmap. Critically, they document that most existing AI tools are trained on human clinical isolates, that geographic bias in reference databases disadvantages the low- and middle-income countries where AMR burden is highest, and that no AI-guided phage therapy has yet been validated in commercial livestock.

## Conclusions

The twelve contributions in this Research Topic demonstrate the rapid evolution of veterinary phage therapy from proof-of-concept studies toward precision antimicrobial interventions. Collectively, they expand the repertoire of phages and phage-derived antimicrobials against major bacterial pathogens in livestock and aquaculture while highlighting the critical role of genomics, microbial ecology, and evolutionary biology in optimizing phage discovery and therapeutic design. Together, these studies underscore that successful phage therapy requires integrating pathogen genomics, host–phage interactions, standardized experimental frameworks, and computational approaches to support evidence-based phage selection. They further emphasize that harmonized regulatory pathways, representative genomic datasets, and artificial intelligence will be essential to accelerate the translation of precision phage therapy into sustainable antimicrobial stewardship strategies within a One Health framework.
